# Effects of heart failure and coronary artery disease on erectile dysfunction: a two-sample mendelian randomization study

**DOI:** 10.1186/s12894-023-01335-1

**Published:** 2023-10-14

**Authors:** Kaiyang Shao, Weikang Chen, Yaling Li, Huiyan Zheng, Ruying Hu, Jianqiao Zhang, Ting Sun

**Affiliations:** 1 The Second Affiliated Hospital of Zhejiang University School of Medicine, 88 Jiefang Road,Shangcheng District Hangzhou, 310009 Hangzhou, Zhejiang China; 2 Women’s Hospital, School of Medicine, Zhejiang University, Hangzhou, Zhejiang China

**Keywords:** Mendelian randomization, Heart failure, Coronary artery disease, Erectile dysfunction, Causal association

## Abstract

**Background and aims:**

There are no clear conclusions as to whether heart failure (HF) and coronary heart disease (CAD) increase the risk of erectile dysfunction (ED).In our study, we used Mendelian randomization (MR) analysis to discover a causal relationship between HF, CAD and ED.

**Methods:**

Single nucleotide polymorphisms (SNPs) associated with HF, CAD and ED were obtained from the MRC IEU Open Genome-Wide Association Study (GWAS) database.After a series of screenings, the remaining SNPs were selected as instrumental variables (IVs) for HF and CAD for MR analysis to assess the relationship between genetically predicted HF or CAD and the pathogenesis of ED.Among them, we used the random-effects inverse variance weighted (IVW) method as the primary analysis method.Finally, Cochran’s q-test, funnel plots, MR-Egger regression, Leave-one-out method and MR-PRESSO were used for sensitivity analysis.

**Results:**

In the IVW method, there was no significant causal relationship between genetically predicted HF and CAD and the incidence of ED.(HF: OR = 1.17, 95% CI 0.99–1.39; p = 0.074;CAD: OR = 1.08, 95% CI 0.99–1.17, p = 0.068)。The results of sensitivity analyses supported our conclusion that no horizontal pleiotropism was found.

**Conclusion:**

This study did not find a causal relationship between HF or CAD and ED in European populations, which requires further in-depth research.

**Supplementary Information:**

The online version contains supplementary material available at 10.1186/s12894-023-01335-1.

## Introduction

Erectile dysfunction (ED) is one of the most common disorders in men and is defined as the long-term inability to achieve or maintain a penile erection. Erectile dysfunction is very important for the well-being and health of men because it not only affects the individual but also causes stress on the lifestyle and relationship of the couple [1].In the United States, at least 12 million men between the ages of 40 and 79 have ED [2].There are many risk factors for ED, including age, coronary artery disease, obesity, smoking, depression, hypertension, previous pelvic surgery, and spinal cord injury, among other psychological factors [3].

Cardiovascular diseases are the largest contributor to the global burden of non-communicable diseases, accounting for 17.9 million deaths (one-third of all deaths) and 45 per cent of deaths due to non-communicable diseases.In Europe, CVD caused 3.9 million deaths (45% of deaths).With the current decline in CVD mortality and the increasing aging of the population, the number of patients with CVD is increasing [4].Among cardiovascular diseases, the relationship between heart failure (HF) and coronary artery disease (CAD) and ED has been more studied.Some foreign studies show that 74–84% of men with HF have ED [5–8].In addition, most CAD patients are often affected by ED [9–11].Therefore, we often think that there is a strong correlation between HF and CAD and ED.

To the best of our knowledge, the available studies are primarily based on observational epidemiological designs and are susceptible to reverse causation and unmeasured confounding factors,failure to correctly understand the causal relationship between the two diseases [12]。To avoid this, Mendelian randomization (MR) has the advantage of using genetic variation as a tool variable, addressing bias in observational studies and thus providing an alternative way to explore causality [13, 14].In this study, we used the MR approach to investigate the causal relationship between the occurrence of ED in HF and CAD.

## Materials and methods

### Design and participants

This study used a two-sample MR design to detect a potential causal relationship between HF and CAD and ED risk.The hypotheses of the MR study include three conditions:(i)Tool variables (IVs) should be associated with exposure to HF and CAD;(ii)There was no clear correlation between IVs and confounders;(iii)IVs affect the risk of ED only through exposure (HF or CAD) and not through other means [15].Only when all three conditions are met can MR design control for potential confounding factors and provide reliable causal impact estimates, proving causal relationships between the two [16].Data on SNPs’ association with HF, CAD, and ED comes from publicly available large-scale genome-wide association studies (Gwas) and can be downloaded from the MRC IEU Open Gwas dataset.As aggregated data on exposure HF, download at GWAS ID: ebi-a-GCST009541 [17], This GWAS study included 47,309 cases and 930,014 controls;As summary statistics for exposed CAD, download them at GWAS ID: ebi-a-GCST005195 [18], The GWAS study included 122,733 cases and 424,528 controls.Summary, ED-related data as outcome variables are available at GWAS ID: ebi-a-GCST006956 [19],The GWAS study collected 6175 cases and 217,630 controls. All patients and controls were European populations.

In addition, all data from MR is publicly accessible (https://gwas.mrcieu.ac.uk/; Last visited on November 7, 2022).The study waived ethical approval, and all subjects in the original genome-wide association study received informed consent.

### Selection of genetic variants

In this study, we obtained SNPs (p < 5 × 10 − 8) significantly correlated with CAD from GWAS aggregated data [20, 21], At the same time, we relaxed the GWAS p-value threshold for HF to 5 × 10 − 6,In order to obtain the appropriate number of SNPs for subsequent analysis [22].Then, we used the PLINK clumping method to calculate the LD through the two-sample MR package and selected independent SNPs with the following conditions (R2 < 0.001, window size = 10,000 kb) [23], to ensure that all the left IVs for HF and CAD are not in linkage disequilibrium (LD). We estimate the strength of the IVs on the basis of the F statistic. The formula is as follows: F = R2(N-2) (1-R2) (R2: variance of exposure explained by selected instrumental variables; N:sample size) [24]; R2 = 2×EAF× (1-EAF)× beta^2/((2×EAF× (1-EAF)× beta^2) + 2× EAF× (1-EAF)× se× N× beta^2) (beta: effect size for SNP; se: standard error for SNP; N:sample size) [25]. IVs were selected whose F > 10. After harmonizing the SNPs in the data source by effector alleles [26], we discoveryed each instrument SNP in the PhenoScanner GWAS database [27] to assess any prior association (P < 5 × 10 − 8) with possible confounding factors (that is Body mass index and Cardiovascular diseases other than the current study diseases) [28–30] to avoid potential confounding. Finally, the SNPs left were selected as IVs for the following MR test.

### Statistical analysis

In the study, we applied the random-effects inverse-variance weighted (IVW) method as the main analysis to evaluate the casual relation of genetically predicted HF and CAD with the risk of ED [31]. Other methods including MR Egger [32], weighted-median [33], weighted mode [34] and simple mode [35] were also applied. Besides, several sensitivity analyses were carried out to evaluate the strength of the association. First, Cochran’s Q test and funnel plots were performed to assess the heterogeneity [36]. Second, we applied MR Egger regression to recognize the existence of directional pleiotropy by calculating whether the intercept was statistically away from zero [32]. Third, we used the Leave-one-out method to verify the robustness of the findings [37]. Fourth, in order to detect possible outliers, we apply the MR pleiotropy residual sum and outlier (MR-PRESSO) test [38]. We used odds ratios (ORs) with their 95% confidence intervals (CIs) to present the associations between HF and CAD and risk of ED and applied RStudio (version 2022.02.3) with ‘TwoSampleMR’ and ‘MRPRESSO’ to perform MR analyses. In this study, p < 0.05 was considered a statistically significant difference.

## Results

### Genetically predicted HF on ED

**Fig. 1:**
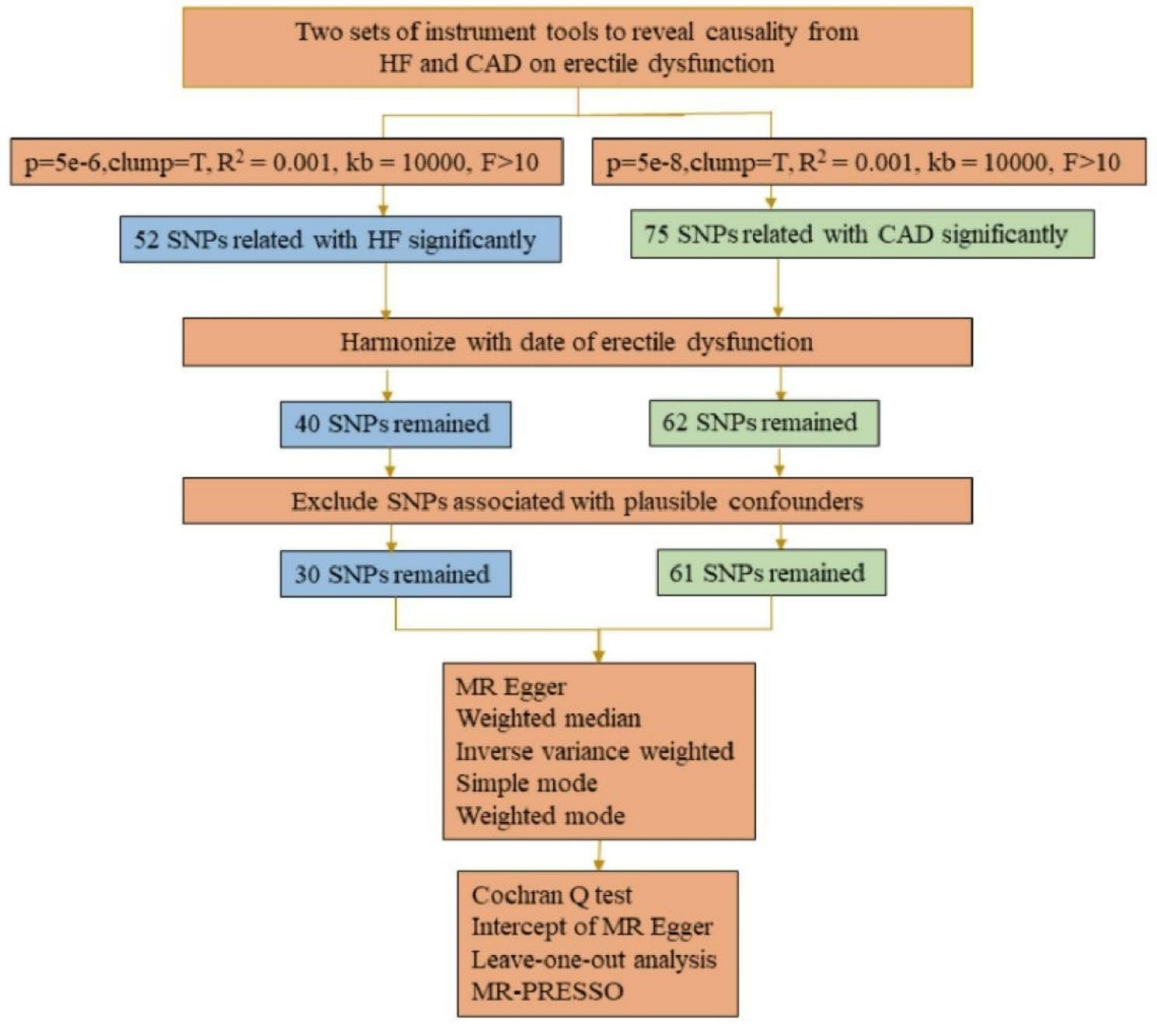
Workflow of Mendelian randomization study revealing causality from HF and CAD on erectile dysfunction. Abbreviations: HF, heart failure; CAD, coronary artery disease; SNP, single-nucleotide Polymorphisms; MR, Mendelian randomization; MR-PRESSO, MR Pleiotropy Residual Sum and Outlier methods

**Fig. 2:**
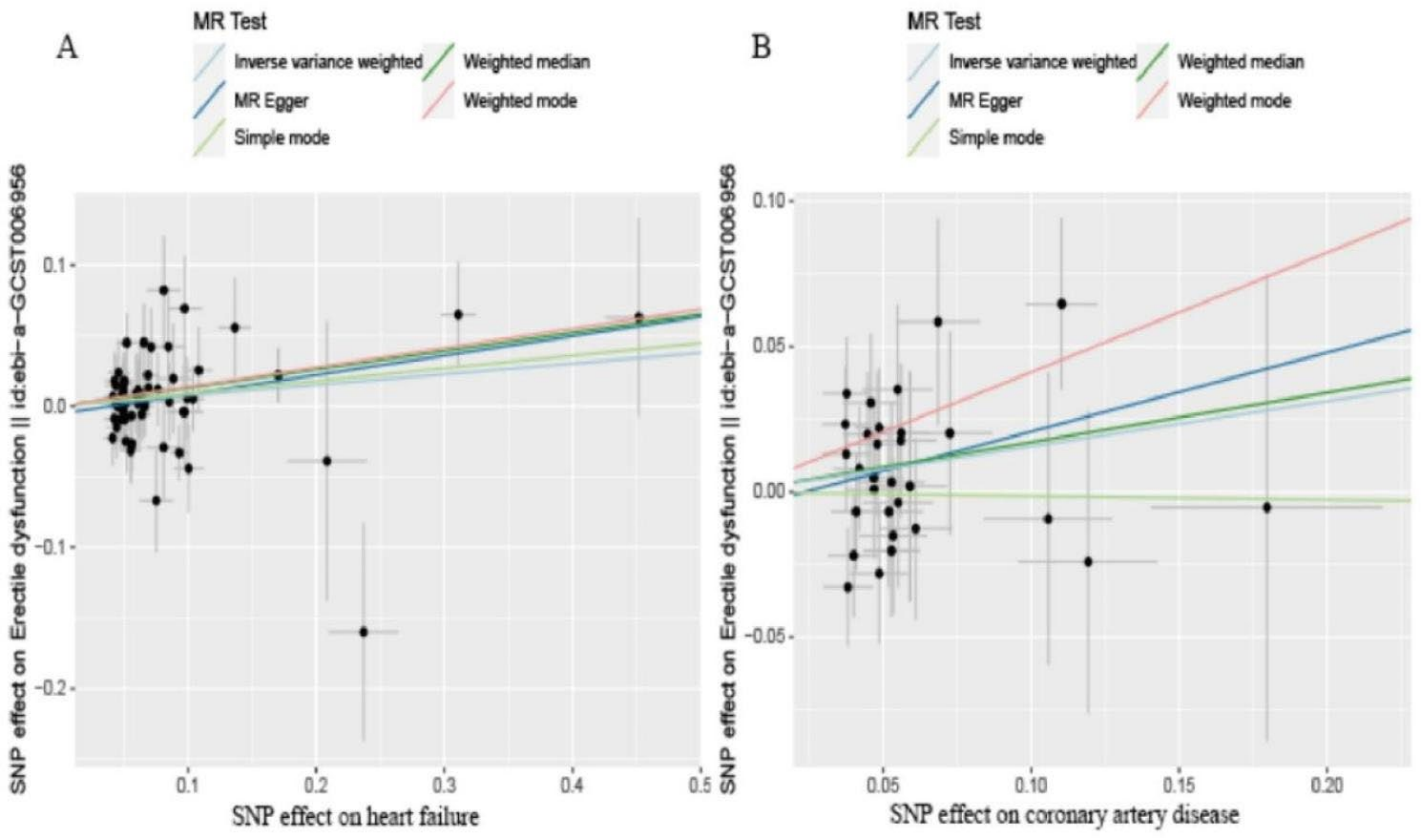
The causality of heart failure (**A**) and coronary artery disease (**B**) on Erectile dysfunction risk. The slope represents the magnitude of the causal effect. Abbreviations: MR, Mendelian randomization

After the above selection (the specific flow chart is shown in Figs. 1), 30 IVs were left, accounting for approximately 2.6% of the observed variance of hf (the F-statistics range from 53.0 to 262.5) (Supplementary Table 1). Genetically predicted HF was not related to ED (OR = 1.17, 95% CI 0.99–1.39; p = 0.074) in the IVW analyses (Fig. 2A). The consistent results were obtained in the weighted median approaches (OR = 1.19, 95% CI = 0.93–1.51, p = 0.164), weighted mode approaches (OR = 1.51, 95% CI = 0.87–2.62, p = 0.154), simple mode (OR = 0.99, 95% CI = 0.56–1.73, p = 0.963) and MR-Egger regression (OR = 1.31, 95% CI = 0.76–2.26, p = 0.333) (Fig. 2B). There was no heterogeneity found by a Cochran Q test (P = 0.731 of MR-Egger; p = 0.766 of IVW) (Table 1) and funnel plots (Supplementary Fig. 1). The MR-Egger intercept did not deviate significantly from zero with a p-value of 0.661(Table 1). The leave-one-out test showed that there were no significant differences (Supplementary Fig. 2) and the MR-PRESSO test did not find any outliers.

**Table 1:** Pleiotropy tests and heterogeneity of MR

	Pleiotropy test			Heterogeneity test					
				MR Egger			IVW		
	egger_intercept	se	pval	Q	Q_df	Q_pval	Q	Q_df	Q_pval
**HF**	-0.006	0.015	0.661	23.033	28	0.731	23.229	29	0.766
**CAD**	-0.005	0.006	0.387	52.020	59	0.728	52.778	60	0.734

Abbreviations: HF: Heart failure; CAD: Coronary artery disease; IVW, inverse variance weighted; MR, Mendelian randomization

### Genetically predicted CAD on ED

After the above selection (the specific flow chart is shown in Figs. 1), 61 IVs were left, accounting for approximately 10.9% of the observed variance of CAD and all the F-statistics were above 10, ranging from 66.6 to297.5 (Supplementary Table 2). Genetically predicted was not related to risk of ED (OR = 1.08, 95% CI 0.99–1.17, p = 0.068) in the IVW analyses (Fig. 2B). Meanwhile, similar results were discovered by weighted mode approaches (OR = 1.15, 95% CI = 0.99–1.33, p = 0.074), simple mode (OR = 1.09, 95% CI = 0.85–1.40, p = 0.481) and MR-Egger regression (OR = 1.15, 95% CI = 0.98–1.35, p = 0.101). No heterogeneity was found in the study with a Cochran Q-test (P = 0.728 of MR-Egger; p = 0.734 of IVW) (Table 1) and funnel plots (Supplementary Fig. 3). The MR Egger intercept did not deviate significantly from zero with a p-value of 0.387 (Table 1). The leave-one-out test also generally support our results while we removed a single SNP and applied the MR analysis again, demonstrating our results’ robustness (Supplementary Fig. 4). By using the MR-PRESSO test, Outliers are not found, verifying the absence of unknown pleiotropic effects of the genetic instruments.

## Discussion

In this study, we used a two-sample MR analysis method to investigate the causal relationship between HF or CAD and ED risk.However, in our results, we did not find a significant causal relationship between HF or CAD and ED.

To our knowledge, the relationship between HF and ED is currently unclear.However, many scholars believe that HF patients are often accompanied by ED, and there is a certain correlation between the two.An observational study by Andrea Crafa et al. showed that arterial ED is strongly associated with cardiovascular risk [39].In a cross-sectional study, Zeighami Mohammadi et al. studied 100 men with systolic heart failure (HF), by filling in the International Erectile Function Index-5 item (IIEF-5), the Minnesota Heart Failure Living Questionnaire (MLHFQ) assesses the extent of its ED and HF, It was found that 80% of patients with HF had ED, of these, 36% suffered from severe erectile dysfunction [5]. In the study of Medina et al. [6], Schwarz et al. [7]and Rastogi et al. [8] also found that patients with HF had a higher prevalence of erectile dysfunction in 74%, 84%, and 75%, respectively.

Many researchers believe that the mechanisms of ED in HF patients are diverse, such as endothelial dysfunction, reduced exercise tolerance, heart drugs, and HF-related hypogonadism [40]. (1) Endothelial dysfunction.This pathology is closely related to HF, which may cause reduced NO production and limited vasodilation, which in turn causes ED [41, 42]. (2) Exercise tolerance disorders.Depending on the severity of cardiac function, exercise tolerance in people with HF decreases to varying degrees, from limitations in physical exertion to limitations in basic activities of daily living. Therefore, although the physical work associated with sexual activity is relatively moderate [43], but some people with HF, especially those with NYHA class III-IV, may not be able to afford the energy expenditure of sex.A study by Basile L et al. showed that the vasodilatory effect of sildenafil improved athletic performance. This also reflects the correlation between HF and ED [44]0.3. Cardiac medication:Because HF-modifying drugs have multiple vascular, metabolic, and neurohumoral effects, many cardiovascular drugs (such as thiazide diuretics, β blockers, and lipid-lowering drugs) have been found to negatively affect erectile function [45, 46]. 4. Anabolic disorders:Metabolic imbalances are typical of patients with HF and often lead to increased catabolism and cardiac cachexia.Anabolic hormones, including insulin-like growth factor-1 (IGF-1), dehydroepiandrosterone sulfate (DHEA-S), and total testosterone (TT), could enhance exercise tolerance in healthy men.A decrease in these hormones can lead to a decrease in exercise capacity.Antonio Aversa et al. reported that decreased androgen production leads to the development of late-onset hypogonadism, which is characterized by erectile dysfunction (ED) and hypogonadism [47].Many studies have shown an increased prevalence of anabolic disorders in HF. Jankowska et al. evaluated 208 patients with HF of various etiologies and found that TT and DHEA-S levels were inversely correlated with NYHA grades [48]. The common clinical manifestation of anabolic disorders and hypogonadism is ED.To further investigate the direct causal relationship of HF for ED, we used the Mendelian randomization method and found that HF did not directly contribute to the risk of developing ED in our study.Therefore, the relationship between HF and ED needs further study.

Similarly, the relationship between CAD and ED is not clear.In a cross-sectional observational study, Kałka et al. [9]recruited 751 rehabilitative CAD patients in five cardiac rehabilitation centers, and found that ED was present in 568 (75.63%) of the patients. In addition, many scholars believe that the coexistence of clinically obvious CVD and ED symptoms is a common phenomenon [10, 11]. Regarding the co-existence mechanism between the two, endothelial dysfunction is considered to be a common risk factor for both diseases, causing the occurrence of both diseases [10, 49]. However, no causal studies have been conducted on the relationship between the two.In our study, we further explored whether CAD directly causes ED.Finally, our results suggest that CAD does not directly cause ED.Further confirmation of the coexistence between the two diseases may be caused by common risk factors, but further confirmation is needed.

The MR study design is one of the greatest strengths of this study. This approach can reverse causality inherent and minimize residual confounding in observational studies. Besides, it can allow us to discovery potential causal relationships between erectile dysfunction and CAD or HF. The study can further support the results through other secondary analytical approaches and sensitivity analyses, increasing the reliability of our conclusions. In addition, we extracted the instrumental variables from the most recent GWAS available with confidence to minimize weak instrumental bias.

However, there were some several limitations. First, the data from GWASs of this study came from European, so that the similar study should be investigated in other populations. Second, there are different subtypes of ED (non-vascular or vascular), which were not distinguished in this study. Subsequent studies could be devoted to ED analysis of different subgroups. For example, ED was divided into two groups, non-vascular and vascular, and MR Was used for analysis. The conclusions of each group were compared to see whether there would be a positive result for vascular ED.Similarly, a targeted study on the different types of HF (high ejection fraction or low ejection fraction), could provide further information about the association between these conditions, to formulate predictive parameters of severity or even response to therapy.Thirdly, only 2.6% of the observed variance in HF was explained by IVs, so the statistical power may be insufficient. Therefore, for this negative result, we need to interpret it with caution to avoid drawing this conclusion due to insufficient power.

## Conclusion

This is the first study to explore the causal relationship between HF, CAD and ED. We did not find a causal relationship between HF or CAD and ED in European populations, which requires further in-depth research to verify.

### Electronic supplementary material

Below is the link to the electronic supplementary material.

Supplementary Material 1

## Data Availability

Data on heart failure and coronary artery disease was provided by Shah et al. (2020) and van der Harst et al. (2017). Data on erectile dysfunction was provided by Bovijn et al. (2019). The datasets generated and analysed during the current study are available in the IEU open gwas project [https://gwas.mrcieu.ac.uk/datasets/], and the GWAS ID are ebi-a-GCST009541, ebi-a-GCST005195 and ebi-a-GCST006956, respectively.
