# Supplementary material for: Effects of heart failure and coronary artery disease on erectile dysfunction: a two-sample mendelian randomization study
Source: BMC Urol. 2023 Oct 14;23:163. doi: 10.1186/s12894-023-01335-1 (PMC10576338; doi:10.1186/s12894-023-01335-1)

Supplementary table 1: instrumental variables for Heart failure.

| SNP | chr | positon | EA | OA | EAF | beta | se | p | R^2 | F |
| --- | --- | --- | --- | --- | --- | --- | --- | --- | --- | --- |
| rs10150022 | 14 | 89858683 | G | A | 0.7168 | -0.0419 | 0.0087 | 1.35E-06 | 0.000713 | 314.0749 |
| rs10459012 | 11 | 55091574 | A | C | 0.2121 | 0.0458 | 0.0095 | 1.49E-06 | 0.000701 | 308.924 |
| rs10882816 | 10 | 98311190 | T | G | 0.3033 | -0.0447 | 0.0085 | 1.35E-07 | 0.000844 | 372.1384 |
| rs11722972 | 4 | 69897984 | G | T | 0.1693 | -0.0519 | 0.0114 | 4.94E-06 | 0.000758 | 333.8638 |
| rs11745324 | 5 | 1.37E+08 | A | G | 0.24 | -0.0528 | 0.0095 | 2.34E-08 | 0.001017 | 448.2692 |
| rs117925145 | 7 | 12866711 | G | A | 0.0167 | 0.1797 | 0.0391 | 4.43E-06 | 0.001061 | 467.4808 |
| rs11874705 | 18 | 46508620 | G | A | 0.205 | 0.0469 | 0.0098 | 1.75E-06 | 0.000717 | 315.9239 |
| rs12477245 | 2 | 1.08E+08 | T | C | 0.0384 | 0.1192 | 0.0236 | 4.43E-07 | 0.001049 | 462.5285 |
| rs12940636 | 17 | 53400110 | C | T | 0.3473 | -0.0381 | 0.0083 | 4.71E-06 | 0.000658 | 289.9737 |
| rs17042102 | 4 | 1.12E+08 | A | G | 0.1221 | 0.1103 | 0.0121 | 5.71E-20 | 0.002608 | 1151.464 |
| rs17496249 | 2 | 37102249 | G | A | 0.447 | -0.0372 | 0.0079 | 2.58E-06 | 0.000684 | 301.4533 |
| rs17617337 | 10 | 1.21E+08 | T | C | 0.2119 | -0.0561 | 0.0095 | 3.65E-09 | 0.001051 | 463.3392 |
| rs2680705 | 17 | 56495584 | C | T | 0.2011 | 0.0486 | 0.0098 | 6.70E-07 | 0.000759 | 334.4345 |
| rs35005436 | 7 | 74134911 | C | T | 0.1604 | 0.0533 | 0.0116 | 4.37E-06 | 0.000765 | 337.1848 |
| rs35054810 | 1 | 2.23E+08 | A | G | 0.0852 | 0.0725 | 0.0143 | 3.76E-07 | 0.000819 | 361.0789 |
| rs4135240 | 6 | 36647680 | C | T | 0.3504 | -0.0486 | 0.0084 | 6.84E-09 | 0.001075 | 473.9737 |
| rs55949718 | 6 | 1.18E+08 | T | C | 0.0858 | -0.0685 | 0.0142 | 1.46E-06 | 0.000736 | 324.3648 |
| rs578065 | 17 | 15764507 | G | T | 0.3526 | 0.0408 | 0.0082 | 7.31E-07 | 0.00076 | 334.8959 |
| rs593467 | 1 | 70584460 | A | G | 0.8673 | -0.0548 | 0.0118 | 3.36E-06 | 0.000691 | 304.5833 |
| rs61733868 | 11 | 12261007 | C | T | 0.0413 | -0.1057 | 0.0216 | 1.02E-06 | 0.000885 | 389.9164 |
| rs6922885 | 6 | 79849376 | C | T | 0.5336 | -0.0377 | 0.008 | 2.41E-06 | 0.000707 | 311.7229 |
| rs72844714 | 2 | 1.33E+08 | A | C | 0.1656 | 0.0559 | 0.0121 | 3.88E-06 | 0.000864 | 380.5728 |
| rs73200714 | 8 | 10534097 | A | G | 0.1325 | -0.055 | 0.0118 | 3.37E-06 | 0.000695 | 306.4201 |
| rs7369998 | 2 | 1.26E+08 | A | G | 0.4233 | -0.059 | 0.0126 | 2.90E-06 | 0.0017 | 749.6271 |
| rs7559452 | 2 | 3885011 | G | A | 0.1755 | 0.0468 | 0.0102 | 4.76E-06 | 0.000634 | 279.2796 |
| rs76117960 | 7 | 33441523 | C | T | 0.1465 | 0.0528 | 0.0113 | 2.71E-06 | 0.000697 | 307.1965 |
| rs7766436 | 6 | 22598259 | T | C | 0.2917 | 0.04 | 0.0086 | 3.76E-06 | 0.000661 | 291.3166 |
| rs80087882 | 2 | 2.01E+08 | A | G | 0.1109 | 0.0609 | 0.0125 | 1.17E-06 | 0.000731 | 322.2841 |
| rs9815816 | 3 | 85930582 | C | T | 0.1874 | 0.0479 | 0.0099 | 1.29E-06 | 0.000699 | 307.9111 |
| rs994980 | 10 | 36411104 | T | C | 0.6059 | 0.0375 | 0.0081 | 3.83E-06 | 0.000672 | 295.9143 |

Abbreviations: chr, chromosome; EA, effect allele; OA, other allele; EAF, effect allele frequency.

Supplementary table 2: instrumental variables for coronary artery disease.

| SNP | chr | positon | EA | OA | EAF | beta | se | p | R^2 | F |
| --- | --- | --- | --- | --- | --- | --- | --- | --- | --- | --- |
| rs10131519 | 14 | 100359294 | C | T | 0.182 | 0.049902 | 0.008969 | 2.60E-08 | 0.000741 | 231.1829 |
| rs10793514 | 10 | 44496971 | C | T | 0.6662 | 0.050745 | 0.007328 | 4.40E-12 | 0.001145 | 282.9596 |
| rs11057840 | 12 | 125316055 | C | A | 0.1427 | 0.071246 | 0.010064 | 1.40E-12 | 0.001242 | 206.039 |
| rs11072783 | 15 | 78965966 | A | G | 0.8207 | -0.05596 | 0.009211 | 1.20E-09 | 0.000922 | 225.1091 |
| rs112043140 | 3 | 46585022 | T | C | 0.2234 | 0.046432 | 0.008366 | 2.90E-08 | 0.000748 | 247.8543 |
| rs1148497 | 8 | 102856517 | G | A | 0.2119 | -0.04591 | 0.008407 | 4.70E-08 | 0.000704 | 246.662 |
| rs11591147 | 1 | 55505647 | T | G | 0.017 | -0.23699 | 0.026967 | 1.50E-18 | 0.001877 | 76.89339 |
| rs11601507 | 11 | 5701074 | A | C | 0.0758 | 0.08101 | 0.013518 | 2.10E-09 | 0.000919 | 153.3983 |
| rs11637783 | 15 | 79139000 | C | T | 0.443 | -0.06397 | 0.007035 | 9.70E-20 | 0.002019 | 294.7384 |
| rs11673093 | 19 | 45742094 | A | G | 0.2662 | 0.044446 | 0.008019 | 3.00E-08 | 0.000772 | 258.5864 |
| rs117733303 | 6 | 160922870 | G | A | 0.0187 | 0.451693 | 0.026363 | 8.40E-66 | 0.007488 | 78.6536 |
| rs12212146 | 6 | 161125454 | C | T | 0.0706 | -0.08856 | 0.013938 | 2.10E-10 | 0.001029 | 148.7682 |
| rs12315434 | 12 | 57780936 | C | A | 0.2131 | -0.05046 | 0.008495 | 2.80E-09 | 0.000854 | 244.1064 |
| rs12740374 | 1 | 109817590 | T | G | 0.2203 | -0.10396 | 0.008322 | 8.20E-36 | 0.003713 | 249.1608 |
| rs12930452 | 16 | 75462055 | G | A | 0.5933 | 0.048964 | 0.007104 | 5.50E-12 | 0.001157 | 291.8692 |
| rs1412444 | 10 | 91002927 | T | C | 0.3425 | 0.049455 | 0.007322 | 1.40E-11 | 0.001102 | 283.2101 |
| rs146534110 | 6 | 160578069 | T | G | 0.0132 | 0.208513 | 0.031157 | 2.20E-11 | 0.001133 | 66.55357 |
| rs15285 | 8 | 19824667 | T | C | 0.2877 | -0.04796 | 0.007669 | 4.00E-10 | 0.000943 | 270.3828 |
| rs16986953 | 2 | 19942473 | A | G | 0.0672 | 0.081162 | 0.013412 | 1.40E-09 | 0.000826 | 154.6118 |
| rs17114046 | 1 | 56966350 | G | A | 0.0895 | -0.0966 | 0.011807 | 2.80E-16 | 0.001521 | 175.6226 |
| rs17228058 | 15 | 67450305 | G | A | 0.2365 | -0.05185 | 0.008289 | 4.00E-10 | 0.000971 | 250.1561 |
| rs17263917 | 5 | 9552338 | A | G | 0.1542 | -0.06173 | 0.009819 | 3.20E-10 | 0.000994 | 211.1845 |
| rs17465982 | 1 | 222837939 | A | G | 0.7177 | 0.066123 | 0.007679 | 7.30E-18 | 0.001772 | 270.0152 |
| rs2107595 | 7 | 19049388 | A | G | 0.1669 | 0.076585 | 0.009532 | 9.40E-16 | 0.001631 | 217.5311 |
| rs2128739 | 11 | 103673277 | C | A | 0.7188 | -0.06598 | 0.007699 | 1.00E-17 | 0.00176 | 269.3205 |
| rs2140480 | 9 | 110526109 | C | T | 0.6995 | -0.04373 | 0.007818 | 2.20E-08 | 0.000804 | 265.2395 |
| rs2166529 | 2 | 85742175 | T | G | 0.4501 | 0.056371 | 0.007036 | 1.10E-15 | 0.001573 | 294.7288 |
| rs223290 | 6 | 57106712 | C | T | 0.9351 | -0.09727 | 0.014169 | 6.70E-12 | 0.001148 | 146.3419 |
| rs2293251 | 3 | 138124114 | G | T | 0.1541 | 0.061081 | 0.009726 | 3.40E-10 | 0.000973 | 213.1894 |
| rs246600 | 5 | 142516897 | T | C | 0.4813 | 0.043648 | 0.006979 | 4.00E-10 | 0.000951 | 297.1008 |
| rs2649999 | 12 | 121380544 | C | T | 0.6489 | -0.04262 | 0.007546 | 1.60E-08 | 0.000828 | 274.7882 |
| rs28451064 | 21 | 35593827 | A | G | 0.1355 | 0.099283 | 0.010756 | 2.70E-20 | 0.002309 | 192.7832 |
| rs2891168 | 9 | 22098619 | G | A | 0.49 | 0.170449 | 0.006986 | 1.80E-131 | 0.014521 | 296.8006 |
| rs3127580 | 6 | 160710851 | T | C | 0.1445 | 0.085249 | 0.009766 | 2.60E-18 | 0.001797 | 212.3217 |
| rs318719 | 19 | 11496981 | C | T | 0.0791 | 0.07534 | 0.012927 | 5.60E-09 | 0.000827 | 160.4017 |
| rs3741380 | 11 | 65349063 | A | G | 0.4882 | 0.041127 | 0.00697 | 3.60E-09 | 0.000845 | 297.5147 |
| rs3796581 | 4 | 156642884 | G | A | 0.1906 | -0.06867 | 0.009018 | 2.60E-14 | 0.001455 | 229.9398 |
| rs3918226 | 7 | 150690176 | T | C | 0.0791 | 0.097336 | 0.013284 | 2.30E-13 | 0.00138 | 156.0981 |
| rs4280376 | 19 | 17861209 | C | T | 0.1819 | 0.05565 | 0.008981 | 5.80E-10 | 0.000922 | 230.8725 |
| rs4299376 | 2 | 44072576 | T | G | 0.6803 | -0.04536 | 0.007507 | 1.50E-09 | 0.000895 | 276.2323 |
| rs4762479 | 12 | 95495041 | T | C | 0.0742 | -0.08457 | 0.013371 | 2.50E-10 | 0.000983 | 155.0824 |
| rs4803455 | 19 | 41851509 | A | C | 0.4903 | -0.04985 | 0.007006 | 1.10E-12 | 0.001242 | 295.9829 |
| rs4932373 | 15 | 91429287 | C | A | 0.3311 | 0.069068 | 0.007499 | 3.30E-20 | 0.002113 | 276.5078 |
| rs55730499 | 6 | 161005610 | T | C | 0.0736 | 0.310796 | 0.013348 | 6.30E-120 | 0.013172 | 155.3508 |
| rs566818 | 11 | 75158350 | G | A | 0.6871 | -0.04244 | 0.007675 | 3.20E-08 | 0.000775 | 270.1735 |
| rs604723 | 11 | 100610546 | C | T | 0.7281 | 0.049726 | 0.007884 | 2.80E-10 | 0.000979 | 263.0237 |
| rs6460942 | 7 | 12420989 | C | T | 0.1368 | -0.05942 | 0.010286 | 7.60E-09 | 0.000834 | 201.598 |
| rs6511720 | 19 | 11202306 | T | G | 0.1123 | -0.10845 | 0.0107 | 3.90E-24 | 0.002345 | 193.7867 |
| rs685031 | 6 | 31881731 | A | G | 0.6333 | 0.045558 | 0.00723 | 3.00E-10 | 0.000964 | 286.8073 |
| rs6905288 | 6 | 43758873 | A | G | 0.5719 | 0.041279 | 0.007026 | 4.20E-09 | 0.000834 | 295.1151 |
| rs701145 | 3 | 154054799 | C | T | 0.8561 | 0.06562 | 0.009823 | 2.40E-11 | 0.001061 | 211.0886 |
| rs7209460 | 17 | 2048713 | T | C | 0.6918 | 0.052175 | 0.007622 | 7.60E-12 | 0.001161 | 272.045 |
| rs72934535 | 2 | 203968973 | C | T | 0.1074 | 0.100422 | 0.011558 | 3.70E-18 | 0.001934 | 179.4062 |
| rs7412 | 19 | 45412079 | T | C | 0.0833 | -0.13673 | 0.012741 | 7.30E-27 | 0.002855 | 162.747 |
| rs7502499 | 17 | 47490102 | A | G | 0.319 | 0.052564 | 0.007548 | 3.30E-12 | 0.0012 | 274.7103 |
| rs7678555 | 4 | 120909501 | C | A | 0.2931 | 0.045665 | 0.007745 | 3.70E-09 | 0.000864 | 267.7254 |
| rs77347777 | 3 | 52848207 | T | C | 0.113 | -0.06081 | 0.011046 | 3.70E-08 | 0.000741 | 187.727 |
| rs8003602 | 14 | 100148961 | C | T | 0.7468 | 0.05522 | 0.007965 | 4.10E-12 | 0.001153 | 260.3478 |
| rs9337951 | 10 | 30317073 | A | G | 0.3448 | 0.050146 | 0.007618 | 4.60E-11 | 0.001136 | 272.205 |
| rs9349379 | 6 | 12903957 | G | A | 0.4162 | 0.093244 | 0.007149 | 6.90E-39 | 0.004225 | 290.0598 |
| rs9554448 | 13 | 98859722 | C | T | 0.1047 | -0.06294 | 0.011456 | 3.90E-08 | 0.000743 | 181.0098 |

Abbreviations: chr, chromosome; EA, effect allele; OA, other allele; EAF, effect allele frequency.

Supplementary figure 1: Funnel plot to assess the heterogeneity of heart failure.


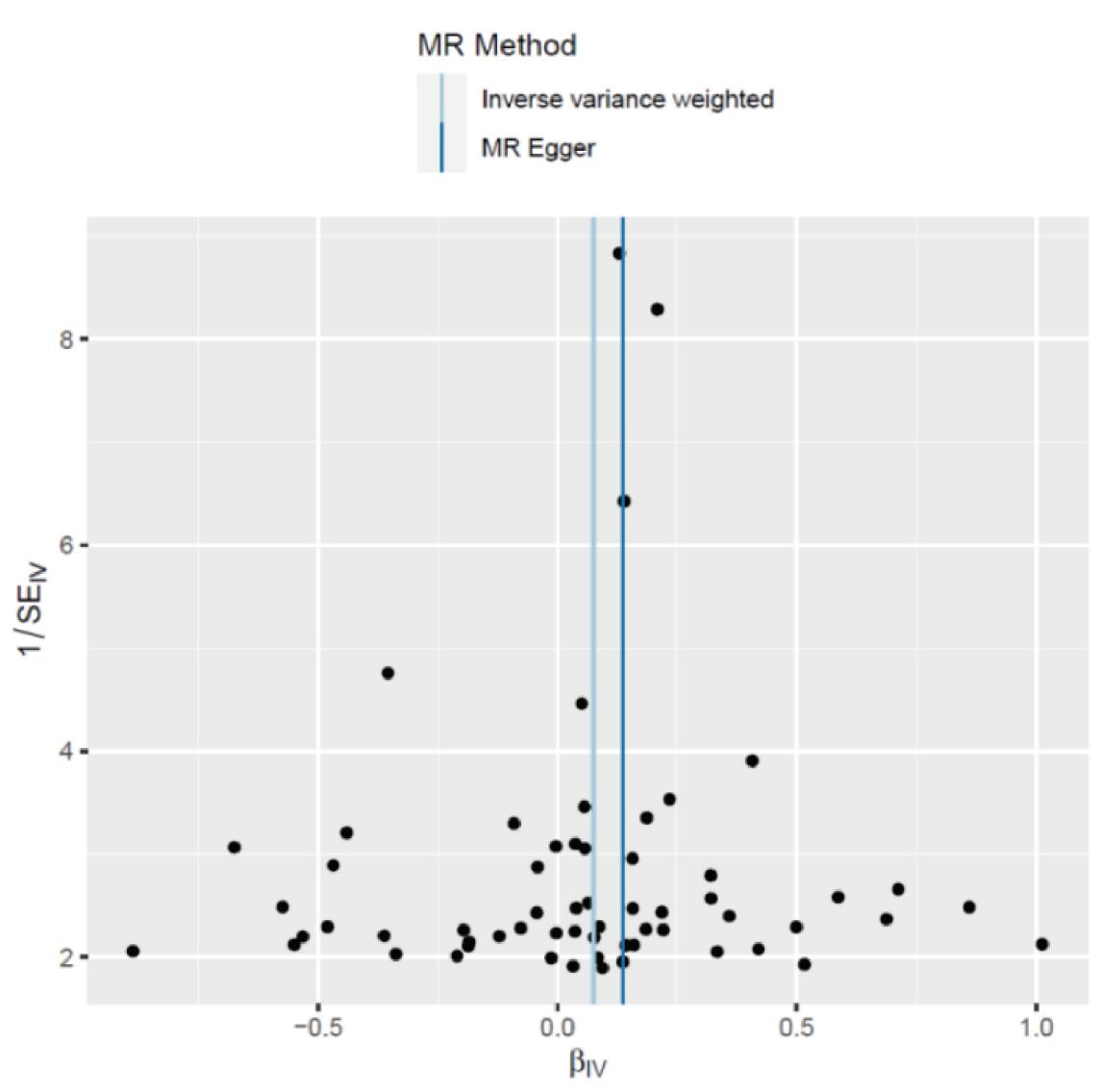


Supplementary figure 2: Leave-one-out analysis of the effect of heart failure on Erectile Dysfunction. Abbreviations: MR, Mendelian randomization.


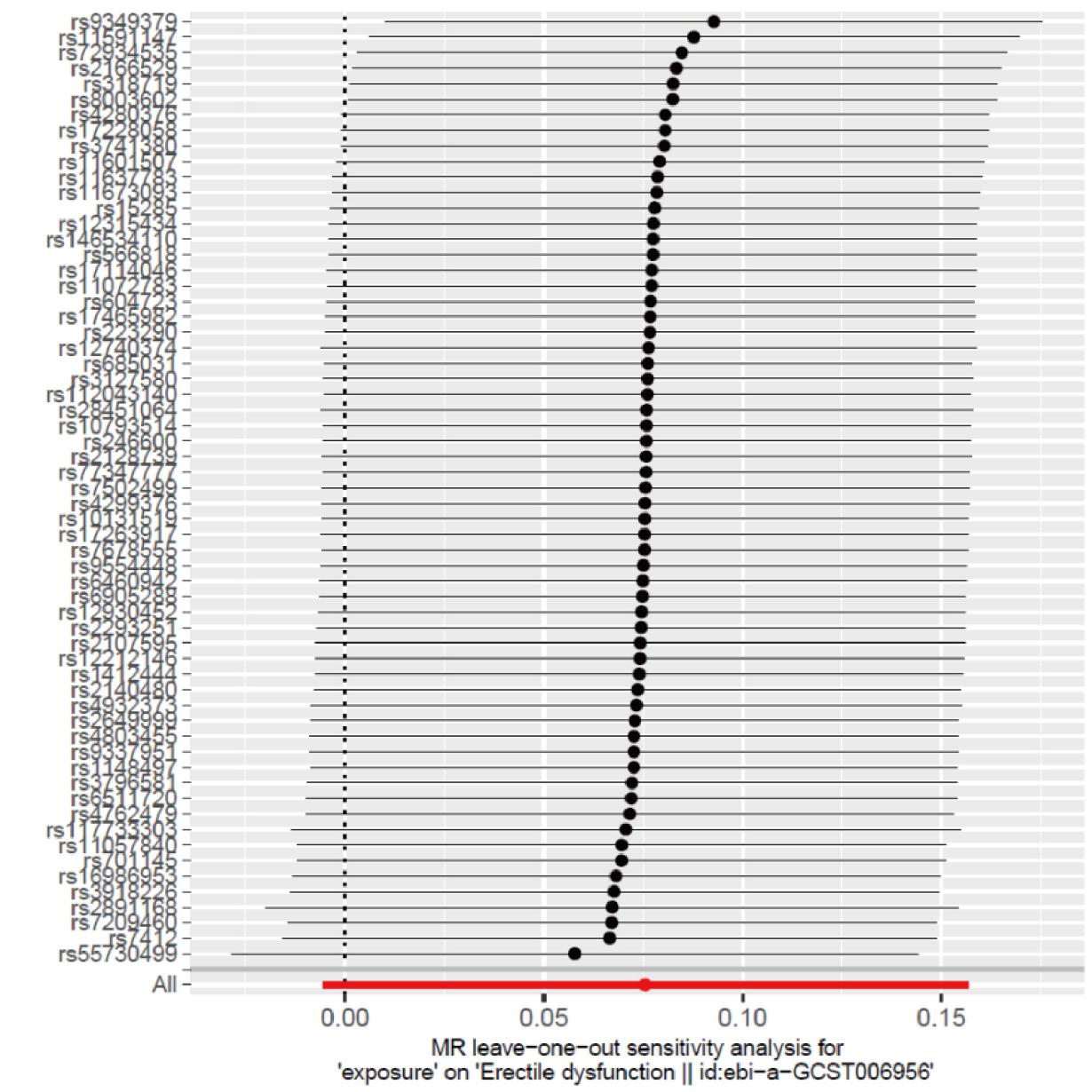


Supplementary figure 3: Funnel plot to assess the heterogeneity of coronary artery disease.


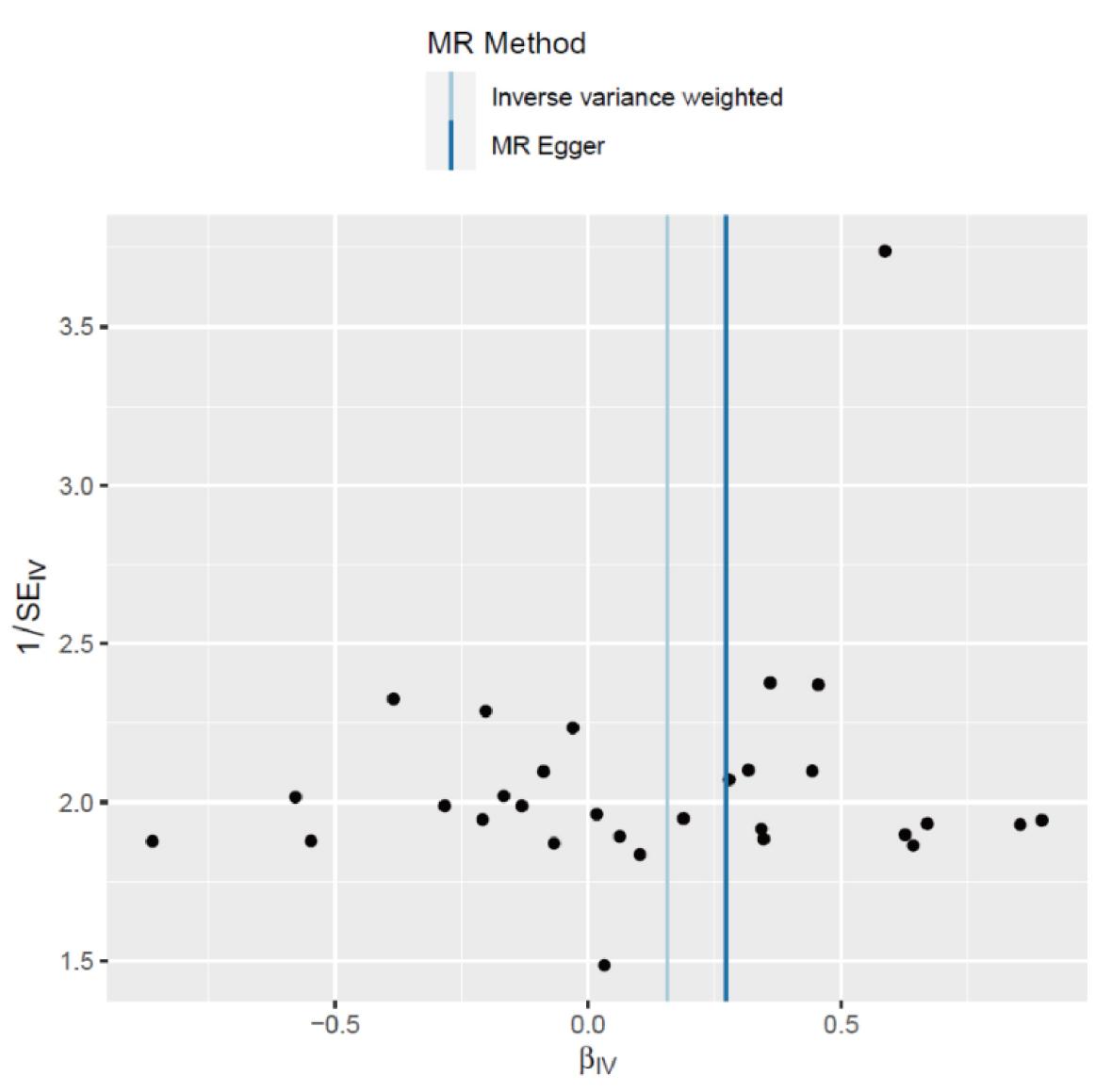


Supplementary figure 4: Leave-one-out analysis of the effect of coronary artery disease on Erectile Dysfunction. Abbreviations: MR, Mendelian randomization.


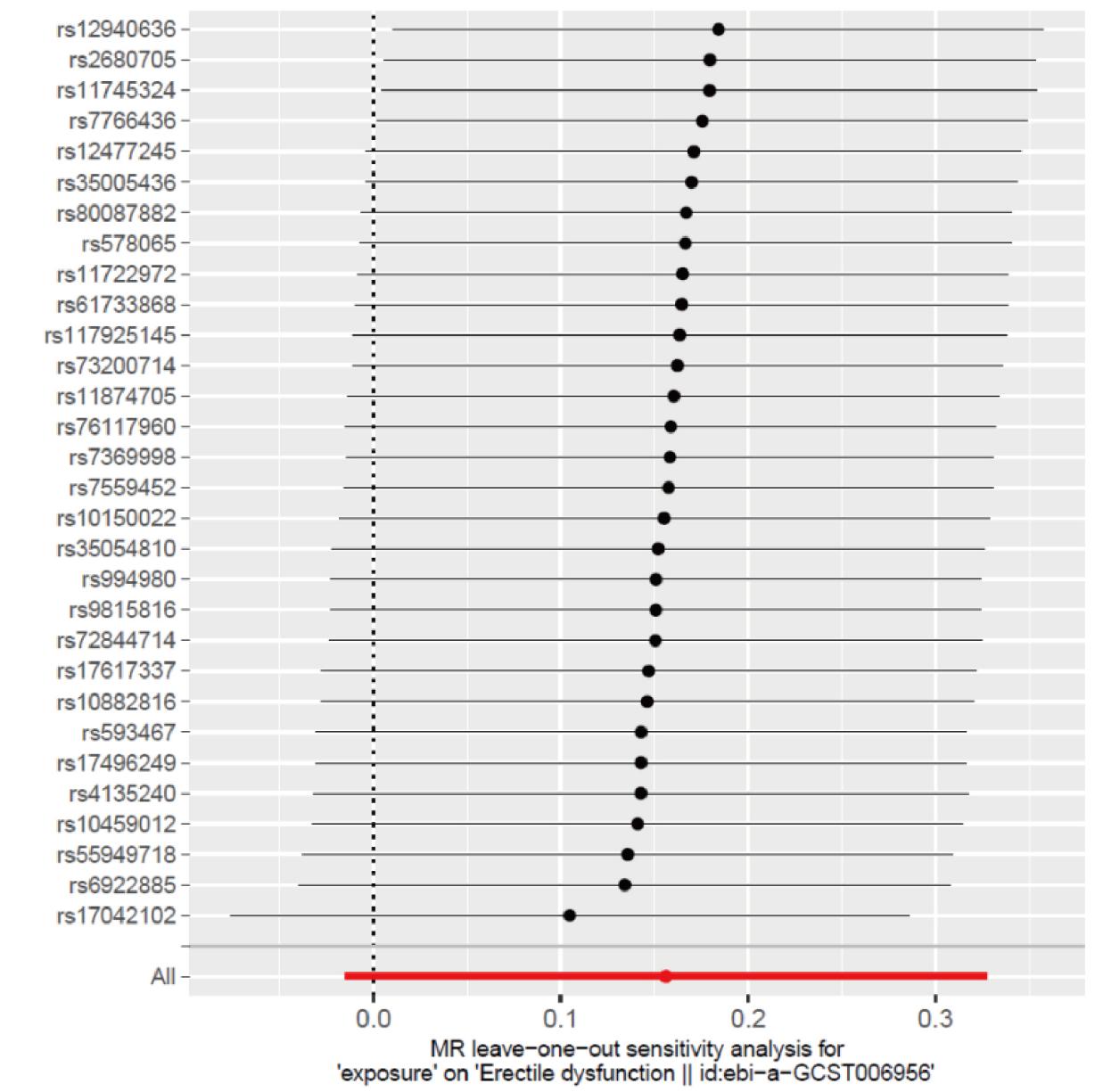

Supplement: Supplementary file 1 — Supplementary Material 1 [file 12894_2023_1335_MOESM1_ESM.docx]
